# RNA Sequencing Reveals Rice Genes Involved in Male Reproductive Development under Temperature Alteration

**DOI:** 10.3390/plants10040663

**Published:** 2021-03-30

**Authors:** Sudthana Khlaimongkhon, Sriprapai Chakhonkaen, Keasinee Tongmark, Numphet Sangarwut, Natjaree Panyawut, Thiwawan Wasinanon, Kannika Sikaewtung, Samart Wanchana, Chareerat Mongkolsiriwatana, Julapark Chunwonges, Amorntip Muangprom

**Affiliations:** 1 Center for Agricultural Biotechnology, Kasetsart University, Kamphaeng Saen Campus, Kamphaeng Saen 73140, Thailand; suthana@yahoo.com (S.K.); julapark@gmail.com (J.C.); 2 Center of Excellence on Agricultural Biotechnology: (AG-BIO/PERDO-CHE), Bangkok 10900, Thailand; 3 National Center for Genetic Engineering and Biotechnology, National Science and Technology Development Agency, Pathum Thani 12120, Thailand; sriprapai.cha@biotec.or.th (S.C.); keasinee.ton@biotec.or.th (K.T.); numphet.san@biotec.or.th (N.S.); natjaree.pan@biotec.or.th (N.P.); thiwawan.was@biotec.or.th (T.W.); kannika.sik@ncr.nstda.or.th (K.S.); samart.wan@biotec.or.th (S.W.); 4 Division of Genetics, Faculty of Liberal Arts and Science, Kasetsart University, Nakhon Pathom 73140, Thailand; faascrp@ku.ac.th; 5 Department of Horticulture, Faculty of Agriculture, Kasetsart University, Nakhon Pathom 73140, Thailand

**Keywords:** transcriptome, RNA-Seq, anther, TGMS, rice

## Abstract

Rice (*Oryza sativa* L.) is one of the most important food crops, providing food for nearly half of the world population. Rice grain yields are affected by temperature changes. Temperature stresses, both low and high, affect male reproductive development, resulting in yield reduction. Thermosensitive genic male sterility (TGMS) rice is sterile at high temperature and fertile at low temperature conditions, facilitating hybrid production, and is a good model to study effects of temperatures on male development. Semithin sections of the anthers of a TGMS rice line under low (fertile) and high (sterile) temperature conditions showed differences starting from the dyad stage, suggesting that genes involved in male development play a role during postmeiotic microspore development. Using RNA sequencing (RNA-Seq), transcriptional profiling of TGMS rice panicles at the dyad stage revealed 232 genes showing differential expression (DEGs) in a sterile, compared to a fertile, condition. Using qRT-PCR to study expression of 20 selected DEGs using panicles of TGMS and wild type rice plants grown under low and high temperature conditions, revealed that six out of the 20 selected genes may be unique to TGMS, while the other 14 genes showed common responses to temperatures in both TGMS and wild-type rice plants. The results presented here would be useful for further investigation into molecular mechanisms controlling TGMS and rice responses to temperature alteration.

## 1. Introduction

Rice (*Oryza sativa* L.) is a staple food for nearly half of the world population. An estimate indicates that the world will have 9.3 billion people by the year 2050, while grain production has been declining [1]. Thus, increases in grain yield are necessary to ensure the world food security. Hybrid rice is a successful technology for raising rice yield. Rice hybrids increase yields by 20–30% under unchanged irrigation conditions [2]. Male sterility in the maternal line is needed to facilitate hybrid seed production. Several temperature-sensitive genic male sterile (TGMS) lines are sterile at high temperature (>25 °C) and fertile at low temperature (<23 °C) conditions [3,4]. In TGMS, high temperature affects microspore mother cells (MMC), causing completion of pollen abortion which results in male sterility [5]. Thus, TGMS is a good model to study effects of temperature on male development. The obtained information could be of benefit for the developments of high yielding-hybrid and inbred rice lines under temperature stresses.

Rice cultivars are increasingly exposed to temperature stresses due to ongoing climate change. Cold stress and high temperature stress affect rice growth and productivity. Typical symptoms of plants responding to low temperature are reduction in photosynthesis and stomatal conductivity, accumulation of reactive oxygen species (ROS), changes of proline or glycinebetaine content, and alteration in carbohydrate metabolism [6]. At the reproductive stage, cold stress causes a reduction in yield due to spikelet sterility and abortion. Spikelet sterility caused by low temperature is a result of pollen abortion after microsporogenesis. In addition, low temperature can induce abnormalities in anther development, and late or incomplete ripening [7]. High temperature stress during reproductive development results in reduction in yield and grain quality. High temperature stress at flowering causes spikelet sterility due to abnormal anther dehiscence, glume closure, or impaired pollination and fertilization [8,9]. High temperature stress during grain filling affects final grain weight and abnormalities in starch granules, causing an increase in chalkiness and resulting in lowering grain quality [8,9].

Rice is an exceptional model monocot with known genome sequences, making it a useful tool for studying reproductive development. Male reproductive development or anther development are involved in fertility and sterility in rice. Male sterility is an essential biological process for flowering plants and crop seed production. Male reproductive development involves a number of events [10,11]. Zhang et al. [10] described the cytology of anther development including cell division, differentiation, degeneration of somatic tissues and development of reproductive cells as they form mature pollen grains through meiosis and mitosis. Kalaiyarasi and Vaidyanathan [12] studied cytology to determine the male sterility mechanism in TGMS lines. Their results indicated that pre and post meiotic genetic systems during anther development, and stamen and pistil primordial stages of panicle development, were sensitive to expression of sterility. Genetic backgrounds and certain environmental factors for male sterility and fertility are critical for rice hybrid breeding. Key genes involved in these processes have been identified and studied for function in rice [11,13,14,15,16].

Transcriptomic analysis, the study of transcripts produced at a specific developmental stage or physiological condition, can shed light onto gene function. Results of transcriptomic analysis of developing rice embryos revealed that many genes related to transcriptional regulation, metabolism, signal transduction and nucleic acid replication/processing and expression occurred in the early and middle stages of embryogenesis [17]. Transcriptome profiling has been used to characterize the mechanisms of anther development and male sterility in monocot and dicot plants such as arabidopsis [18], maize [19], cotton [20,21], Chinese cabbage [22], rapeseed [23,24] and watermelon [25]. In rice, Pan et al. [26] studied the transcriptomes of transgenic TGMS rice lines, TGMS-Co27 and wild-type Hejiang 19 (H1493) at high and low temperatures. Findings from this study revealed genes involved in post-transcriptional and translation processes related to thermosensitive male sterility transitions in TGMS-Co27. In addition, transcriptome profiling was investigated under short-day (SD) and long-day (LD) conditions of photoperiod-sensitive male sterile (PGMS) rice, Nongken 58S. The results indicated that male sterility transition in Nongken 58S involved flowering pathways and circadian rhythms. Seventy-three and 128 differentially expressed genes (DEGs) were identified under short-day and long-day conditions, respectively [27]. Furthermore, Yang et al. [28] studied effects of high temperature and soil nitrogen level during rice spikelet development (meiosis stage) using RNA-Seq. The results revealed that rice spikelet fertility was significantly reduced under high temperature but appropriately higher nitrogen levels could mitigate the negative effects of high temperature on spikelet fertility. Their RNA-Seq results showed that the temperature-responsive genes were cytochrome, heat shock protein, peroxidase and ubiquitin, while the nitrogen-responsive genes involved glutamine synthetase, amino acid transporter, pollen development and plant hormones. Their results suggested that high-nitrogen treatment may improve the gene expression levels to moderate aspects of heat stress.

In this study, we compared semithin sections of anthers of a TGMS rice line, IR68301S, grown under fertile and sterile conditions, to identify the critical stage in the sterile condition. In addition, RNA-Seq was used to compare gene expressions in panicles of the TGMS rice line grown under fertile and sterile conditions. The resulting functionally important differentially expressed genes (DEGs) were used for expression analysis by quantitative Reverse Transcription PCR (qRT-PCR) to identify TGMS-related genes and common temperature responsive genes. The results presented here would be useful for further investigation on the molecular mechanisms of rice responses to low and high temperatures, and will help understand the molecular mechanism controlling TGMS in rice.

## 2. Results

### 2.1. Effects of Temperature on Seed Setting Rate

To study effects of temperature on seed setting rate, IR68301S plants were grown in a natural condition until approximately one month before flowering. These plants were then moved to controlled growth rooms at 24, 26, 28, and 32 °C. At 24 and 26 °C, seed setting rates varied from 72.6% to 78.1% and 61.9% to 66.7%, with averages of 75.4% and 64.3%, respectively. Meanwhile, the seed setting rate at 28 °C and 32 °C were 0% (Table 1). These data showed that fertility-inducing temperature of IR68301S was below 28 °C (24–26 °C), and sterility-inducing temperature was above 28 °C (30–32 °C).

### 2.2. Cytology Characterization of Anther Development in IR68301S

**Figure 1:**
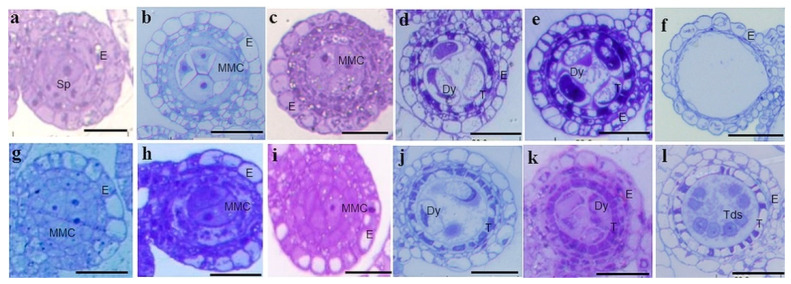
Cytological characterization of anthers. Anthers of IR68301S grown under sterile condition (**a**–**f**), anthers of IR68301S grown under fertile condition (**g**–**l**). E, epidermis; Sp, sporogenous cell; MMC, microspore mother cell; Dy, dyad cell; Tds, tetrads; Msp, microspore parietal cell. Bars = 20 µm.

To identify defects in male reproductive development in IR68301S, spikelets were collected from panicles of the rice plants grown under sterile and fertile conditions and studied in semithin sections. The results showed that there was no detectable difference in the early stages of anther development between sterile and fertile anthers (Figure 1a–d,g–j, respectively). The microspore mother cell (MMC) went through meiosis and formed dyads. At the dyads stage, anthers collected from 13–14 cm panicles of the rice plants grown under sterile condition started to show differences from those collected from 13–14 cm panicles of the rice plants grown under fertile condition. Anthers from sterile condition showed completion of pollen abortion and degradation of tapetum in the tetrad stage, while anthers from fertile condition underwent through the tetrad stage (Figure 1). At the tetrad stage, meiocytes underwent meiosis II, and four newly haploid microspores were generated to produce fertile pollens. Since tapetum cells are a source of nutrients for development of pollen mother cell, the degradation of tapetum leads to sterility of pollen.

### 2.3. Transcriptome Data and Read Assembly

Since semithin sections showed that sterile anthers could develop normally until the dyads stage, the anthers at the dyad stage (panicle size of 13–14 cm) from TGMS rice plants grown under sterile and fertile conditions were selected for transcriptome profiling. Two RNA samples were collected from postmeiotic stage microspore development (dyad to tetrad stage) under fertile and sterile conditions. These samples were used to generate two libraries using Illumina HiSeqTM 2000 platform. The RNA-Seq data after preprocessing and removal of low-quality reads contained a total of approximately 94 million clean reads in both libraries with lengths of 100-bp paired-end reads. The gene expression level was calculated based on the RNA-Seq quantification using Salmon with Nipponbare transcriptome (Phytozome) as reference. The results revealed that approximately 80% of the total clean RNA-Seq reads were successfully mapped onto the rice transcriptome reference for both libraries. The total numbers of genes that were quantified for expression with at least one transcript per million (TPM) were 21,149 and 21,201 for fertile and sterile libraries, respectively. Of the total clean reads from the fertile and sterile libraries, 78.60% and 80.84% were uniquely mapped, 1.54% and 1.51% were mapped to multiple positions and total expressed loci were 31.96% and 32.04%, respectively (Table 2).

### 2.4. Differentially Expressed Genes (DEGs) between Fertile and Sterile Conditions

**Figure 2:**
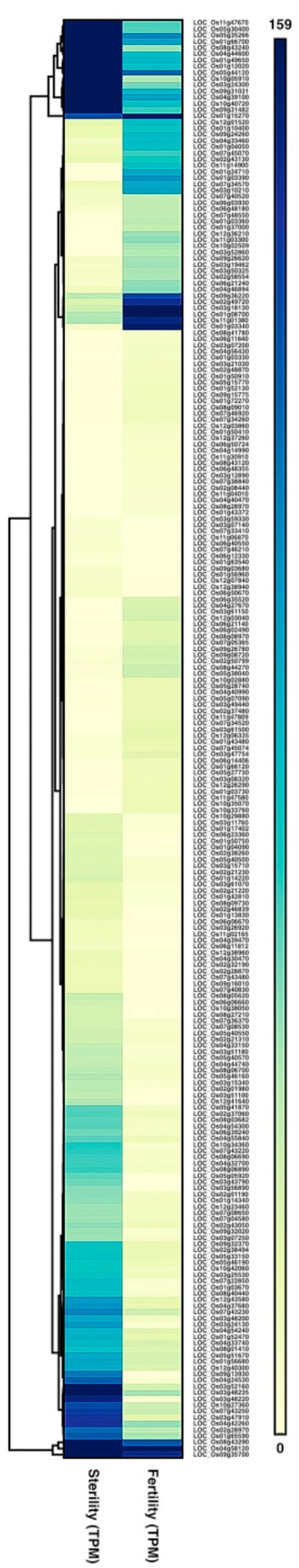
Heat map showing the expression patterns of differentially expressed genes in panicles under the fertile and sterile conditions.

Differentially expressed genes in panicles under fertile and sterile conditions were identified by analyzing the expression profiles of both libraries using the R package DESeq. The results showed that a total number of 18,542 genes were upregulated and 17,113 genes were downregulated in the sterile condition compared to the fertile condition. After filtration with the cut-off threshold adjusted at *p* ≤ 0.05, only 132 and 100 genes were considered significantly up and downregulated DEGs in the sterile condition, respectively. The log2 fold-change values (sterility/fertility) of significantly upregulated genes were in the range of 1.50–9.03 and those of significantly downregulated genes were in the range of (−1.70)–(−9.08). The log2 fold-change values and the normalized transcript per million were presented in the Table S1. The transcriptional response patterns under the fertile and sterile conditions in the panicles of the TGMS line showed complex transcriptional response patterns with several upregulated and downregulated genes, and the levels of the expression alteration varied considerably, as indicated by heatmap analysis (Figure 2).

To understand the functions of these DEGs, all the DEGs were mapped to terms in the GO database and compared to the whole transcriptome background. The DEGs can be categorized into small functional groups in three main categories (biological process, cellular component and molecular function) of the GO classification. A total of 132 up-regulated DEGs were enriched in seven function terms, in which six functional groups were under biological process, and one functional group was under molecular function. The six functional groups under biological process were “anatomical structure morphogenesis” (GO:0009653), “anatomical structure development” (GO:0048856), “multicellular organismal process” (GO:0032501), “developmental process” (GO:0032502), “multicellular organismal development” (GO:0007275) and “cellular component organization” (GO:0016043). The one functional group under molecular function was “lipid binding” (GO:008289). The developmental process, multicellular organismal development and multicellular organismal process were the three most highly enriched GO terms (Table 3). A GO term was considered to be significantly enriched if the *p* ≤ 0.05. There was no GO term enriched in the 100 down-regulated DEGs.

**Figure 3:**
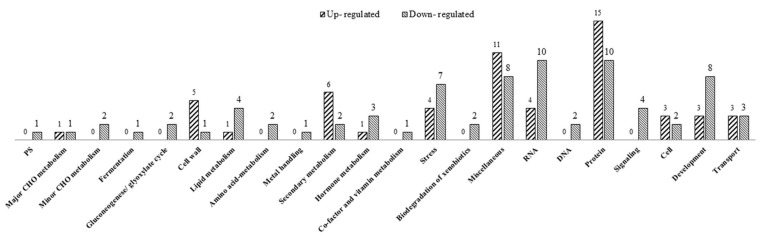
The enriched pathway terms of DEGs based on RNA-Seq data. Numbers on the top of each bar indicated the numbers of genes in that pathway.

MapMan pathway analysis provides classifications for studying the complex biological functions of DEGs. MapMan analysis showed that 57 upregulated DEGs were classified into 12 MapMan functional categories, and 75 upregulated DEGs were classified into the “Not assigned” category. The enriched MapMan pathways for upregulated DEGs were proteins (15 genes) and miscellaneous (11 genes) (Figure 3). The predominant upregulated DEGs in the protein pathways were protein degradation ubiquitin (six genes) and protein glycosylation (four genes). The predominant upregulated DEGs in the miscellaneous pathway were cytochrome P450 (four genes). For downregulated DEGs, 77 genes were classified into 22 MapMan functional categories, and 23 genes were classified into the “Not assigned” category. The enriched MapMan pathways for the down-regulated genes were “Protein” (10 genes), and “RNA” (10 genes). The predominant downregulated DEGs in protein pathway were protein degradation (seven genes). The predominant downregulated DEGs in the RNA pathway were RNA regulation of transcription including transcription factors mainly belonging to WRKY (two genes), Homeobox (two genes), C2C2 (zinc finger family) (two genes), Psudo ARR (one gene) and bHLH (basic Helix-Loop-Helix protein) (one gene).

### 2.5. DEGs Validation

**Figure 4:**
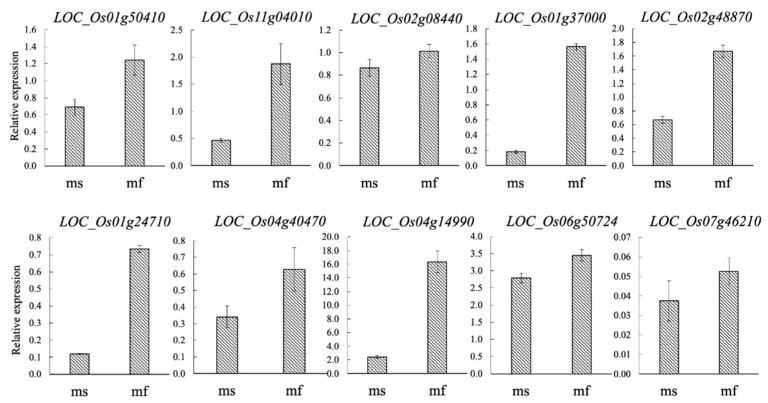
Validation of expression profiles of 10 DEGs by qRT-PCR. MS, sterile temperature; MF, fertile temperature. The relative expression values were normalized to the rice actin gene. The results were obtained from three technical replicates. Error bars indicate standard deviation.

To validate expression profiles of DEGs identified by RNA-Seq, 10 DEGs were randomly selected for expression analysis using qRT-PCR. The expression comparisons were performed between RNA samples isolated from panicles of TGMS rice plants grown under low (fertile) and high (sterile) temperatures. The qRT-PCR results for most of the tested DEGs showed the same trend as that observed in RNA-Seq, except for LOC_Os 07g46210, which showed similar levels of expression in both conditions (Figure 4).

### 2.6. TGMS-Related Genes and Common Temperature Responsive Genes

**Figure 5:**
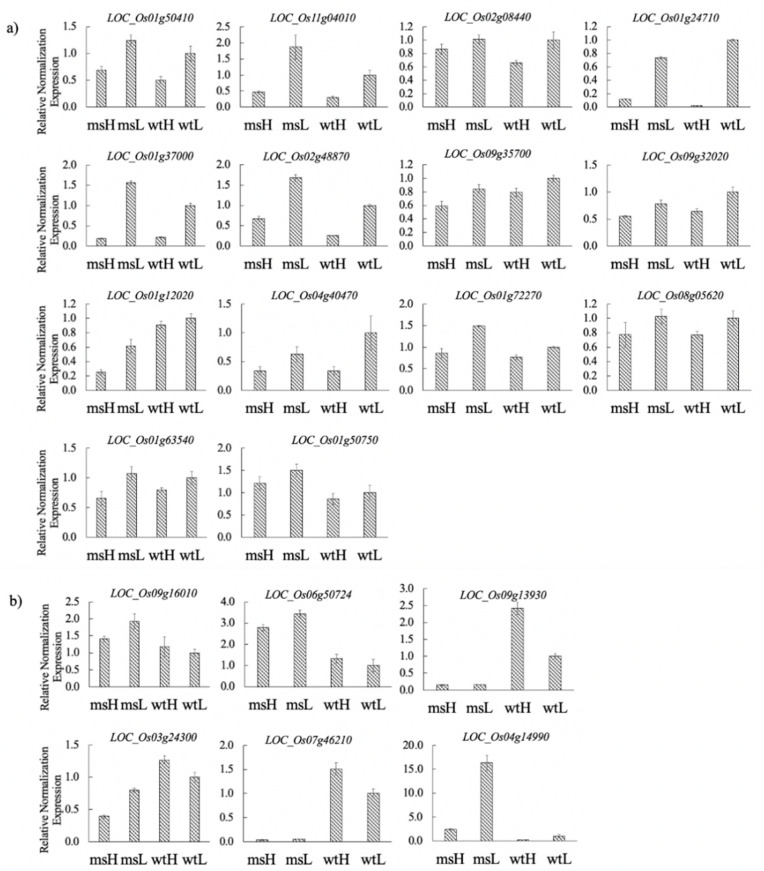
Genes involved in temperature alteration. Common temperature responsive genes (**a**) TGMS-related gene, (**b**) msH, TGMS under high temperature, msL, TGMS under low temperature; wtH, wild-type under high temperature, wtL, wild-type under low temperature. The relative expression values were normalized to the rice actin gene. Error bars indicate standard deviation.

To identify TGMS related genes and common temperature responsive genes, the DEGs selected from enriched pathways, and having potential functional importance, were selected for qRT-PCR expression analysis using RNA samples isolated from panicles of BC3F5 male sterile plants and wild type rice plants grown under low (fertile) and high (sterile) temperatures. The results showed that 20 tested DEGs could be classified into two groups, common temperature responsive genes and TGMS-related genes. In the group of common temperature responsive genes, 14 out of 20 genes showed similar patterns of expressions in TGMS and wild-type rice plants in response to temperature changes by being downregulated in high temperature compared to low temperature conditions (Figure 5a). They were genes encoding for four cytochrome P450, 2 LTPL, ubiquitin, aspartic protease, peptidase, jacalin-like lectin domain, WRKY, protease, and kinase (Table 4). A total of 6 out of 20 genes are possibly TGMS-related genes (Figure 5b). These were genes encoding for 3 LTPL, 2 BURP, and a START domain containing protein (Table 4). Two genes, LOC_Os09g16010(BURP) and LOC_Os06g50724(START) showed downregulation under high temperature compared to low temperature in the TGMS mutant, but in wild-type rice plants these genes showed similar levels of expression in low and high temperature conditions. In addition, the levels of expression in TGMS plants were higher than those in wild type in both conditions. Two genes, LOC_Os07g46210(LTPL2) and LOC_Os09g13930 (LTPL4), showed upregulation under high temperature compared to low temperature in wild-type rice plants, but in the TGMS rice plants these genes showed similar levels of expression by having very low levels of expression in both conditions, and the levels of expression in TGMS plants were much lower than those in wild-type rice plants. LOC_Os03g24300(LTPL1) showed downregulation under high temperature compared to low temperature in the TGMS, but in wild-type rice plants this gene showed upregulation under high temperature compared to low temperature condition. The level of expression in the TGMS was lower than those in wild-type rice plants in both conditions. LOC_Os04g14990(BURP) showed downregulation under high temperature compared to low temperature in the TGMS, but in wild-type rice plants this gene showed very low levels of expression in both conditions. The level of expression in the TGMS was higher than that in wild-type rice plants.

## 3. Discussion

A report from the International Food Policy Research Institute predicted that rice yield could be reduce by climate change by 10–15% in Southeast Asia (Brunei Darussalam, Cambodia, Indonesia, Lao PDR, Malaysia, Myanmar, Philippines, Thailand, Timor-Leste and Viet Nam) [29]. Hybrid rice varieties and related technology are very important in. solving the rice yield shortage for developing countries in this area. The TGMS rice line, IR68301S is sterile at a high temperature (>28 °C) condition but fertile at a low temperature (24–26 °C) condition, facilitating hybrid production. TGMS rice can grow under diverse climatic zones in Southeast Asia. In Thailand, the temperature is favorable for male sterility in IR68301S for most of the year, and the seeds of this TGMS line can be produced in winter during November to January, suggesting that this TGMS line could be used as a maternal line to produce high-yielding hybrids in Thailand and Southeast Asian countries.

In the sterile condition, male development of IR68301S was not different from that in the fertile condition until the dyad stage. At the dyad stage, microspore mother cells proceed through meiosis and form dyads and initiate programmed cell death in tapetum cells. Our results showed that the anthers of IR68301S under high temperature did not go through the tetrad stage, while anthers of IR68301S under the fertile condition developed to the tetrad stage. Semithin sections of anthers of IR68301S grown under the sterile condition showed a critical development event to be at the dyad stage or postmeiotic microspore development, similar to other studies [10,11,12]. The result of transverse sections of anthers of the tms5 rice line, which was grown at 28 °C, showed few or no microspores. In this TGMS rice line, sterile anthers can develop normally until the dyad stage [16]. Kalaiyarasi and Vaidyanathan [12] studied cytological screening of rice TGMS lines, and the result indicated that the sensitive stages of TGMS lines were both the premeiotic phase and post-meiotic stages of panicle development. In broccoli, the cytological difference between flower buds of the CMS line and its maintainer line appeared in the tetrad stage [30]. Tapetum cells are a source of nutrients for development of pollen mother cells. Thus, degradation of tapetum leads to sterility of pollen [22]. Meiosis plays an important role in the life cycle of sexually reproducing organisms and it is a conserved process in eukaryotes. Several genes have been identified to be important for meiosis during rice male reproductive development, such as mutant phenotype of PAIR1, PAIR2 and PAIR3 (HOMOLOGOUS PAIRING ABERATION IN RICE MEIOSIS1,2,3) which are defective in homologous chromosome synapsis causing male and female sterility [31,32,33]. These genes were mapped on chromosome 3, 9, and 10, respectively. Endo et al. [34] revealed that high temperature treatments at the microspore stage induced spikelet sterility. Interestingly, several panicle genes were downregulated under high temperature in our study, similar to other studies [28,34]. According to previous studies, male reproductive development is not only controlled by interactions with environmental factors but also by gene networks [11].

To gain more information on the genes involved in rice fertility and their responses to temperature, the expression profiles of IR68301S under a high temperature (sterile) condition were compared to those under a low temperature (fertile) condition. In our study, RNA-Seq analysis generated 94 million raw reads with lengths of 100 bp. A total of 232 DEGs out of 42,350 expressed genes for sterile and fertile libraries were identified, suggesting that a small number of genes responded to temperature alteration and affected male fertility in TGMS rice. Transcriptome analysis involved in fertility has been reported in many crops. Zhang et al. [20] performed digital gene expression (DGE) analysis of upland cotton genetic male sterility resulting in 1742 DEGs. Of these genes, 916 were upregulated and 826 were downregulated in the sterile compared to fertile library. RNA-Seq was studied in anthers of cotton, male sterile line 1355A and male fertile line 1355B, and 2446 DEGs were identified in three different stages of anther development. The key event for pollen wall formation was after the tetrad stage [35]. RNA-Seq was performed in a watermelon genic male sterile (GMS) line and male fertile line. The results showed that 1259 DEGs were related with male sterility [25]. RNA sequencing analysis in *Brassica napus* (L.) or rapeseed revealed 3199 DEGs between male sterile line and male fertile line [24]. Liu et al. [22] compared the transcriptome profiles of fertile and sterile buds of a Chinese cabbage male sterile line and detected 1013 DEGs, including 907 that were upregulated and 106 that were downregulated. In addition, they found that 475 SEGs (specifically expressed genes; only expressed in fertile or sterile buds) were specifically expressed in fertile buds and six were specifically expressed in sterile buds. The number of DEGs identified in our study was lower than those in other studies, possibly due to the rice genotypes, conditions and analytical methods.

All the DEGs were mapped to terms in the GO database and biological process was the largest group of our GO analysis. Similarly, a total of 179 DEGs were reported to be related to CMS in broccoli (*Brassica oleracea* var. italica) and biological process was the most enriched group of their GO analysis [30]. Our results showed that the major biological functions of DEGs were developmental process, multicellular organismal development and multicellular organismal process, suggesting that these processes could be involved in TGMS.

Mapman analysis revealed that the main pathways of DEGs were protein, RNA and miscellaneous pathways. The protein pathway includes protein degradation ubiquitin E3, cysteine protease, aspartate protease, protein degradation AAA type, and ubiquitin E1. The key expressed genes of the RNA pathway were transcription factors (TF) from several TF families including bHLH (Basic Helix-Loop-Helix), HB (Homeobox), MYB (Myeloblastosis), NAC (No Apica Meristem, ATAF1/2, Cup-shaped Cotyledon2), WRKY, Pseudo ARR and zinc finger family. The key pathway in the miscellaneous category was cytochrome P450. The genes in these pathways could be TGMS-related genes and/or common temperature responsive genes.

Results from qRT-PCR expression analysis of the selected DEGs having functional importance suggested that several DEGs from TGMS panicles could be common temperature-responsive genes for rice plants to cope with temperature alteration during male reproductive development. The ubiquitin-proteasome pathway is highly conserved and found in all eukaryotes [36]. Ubiquitin is one of the temperature-responsive genes involved in rice spikelet development [28]. Our study from RNA-Seq analysis of TGMS’s panicles under low and high temperatures identified three ubiquitin-related genes (LOC_Os09g32020, LOC _Os09g31031, LOC _Os12g01520) as DEGs. In addition, our results on qRT-PCR expression analysis suggested that a ubiquitin-related gene (LOC _Os09g32020), one of the selected DEGs used for qRT-PCR, is a common temperature responsive gene, suggesting that this ubiquitin-related gene helps rice plants, both TGMS and wild type, to cope with temperature alteration during male reproductive development.

Cysteine proteases are ubiquitous hydrolases involved in plant programmed cell dead (PCD). Zhang et al. [37] identified a papain-like cysteine protease, CEP1, which was involved in tapetal PCD and pollen development in *Arabidopsis thaliana*. Aspartic proteases are one of the proteolytic enzymes implicated in protein processing, maturation, and degradation [38]. The putative glycosylphosphatidylinositol (GPI)-anchored aspartic protease genes, A36 and A39 of *Arabidopsis thaliana* are involved in PCD in pollen [38]. Similarly, several protease and protease inhibitor genes were identified as DEGs in our RNA-Seq analysis of TGMS’s panicles. Accordingly, LOC_Os11g04010, LOC_Os01g12012, and LOC_Os09g35700, genes encoding proteases or protease inhibitors were shown to be common temperature-responsive DEGs in panicles using qRT-PCR expression analysis of TGMS and wild type panicles, suggesting their common roles to cope with temperature alteration.

WRKY proteins are a large family of transcription factors. The OsWRKY genes in rice are involved in biotic stress response [39] and abiotic stress such as cold tolerance [40,41]. Expression of WRKY genes is known to be downregulated under cold stress [40], suggesting that this gene plays an important role in rice response to temperature stress. In addition, WRKY protein was reported as a regulator in abiotic stress signaling pathways [42]. Similarly, our RNA-Seq analysis identified the two WRKY genes (LOC_Os02g08440 and LOC_Os05g27730) as DEGs in TGMS panicles. In addition, LOC_Os02g08440 was selected for expression analysis by qRT-PCR, and the result indicated that it is a common temperature-responsive gene.

Biotic and abiotic stress can activate expression of P450 genes [43]. CYP703, an ancient cytochrome P450 specific to land plants, has been reported to be involved in pollen development [44]. Li et al. [45] studied the ω-hydroxylation pathway of fatty acids relying on a cytochrome P450 family, and indicated that formation of ω-hydroxy-palmitic, -palmitoleic, and oleic acids formed by the cytochrome P450 family gene, CYP704B2, play an important role in development of cuticular and sporopollenin polyesters. This pathway is essential in the lipid metabolism/transport required for normal tapetum development during anther and spore development. Certain eukaryotic P450 enzymes undergo post-translational modification including phosphorylation, ubiquitination, glycosylation and nitration [46]. Phosphorylation of CYP3A4 leads to formation of CYP3A4-ubiquitin form in microsomal membranes and enhances protein degradation [47]. Yang et al. [28] reported RNA sequencing of the rice spikelet at the meiosis stage in response to temperature interacting with nitrogen, and found that many cytochrome P450 genes were temperature-responsive genes. Results from Yang’s experiment revealed that high temperature decreased rice spikelet fertility, and numerous spikelet genes involved in male development were mainly downregulated under high temperature. Our results on expression analysis of DEGs using qRT-PCR suggested that four cytochrome P450 genes: LOC_Os04g40470, LOC_Os01g72270, LOC_Os08g05620 and LOC_Os01g63540 are common temperature-responsive genes, and they were downregulated under high temperature in TGMS and wild type rice plants. This agreed well with a previous report that most of the P450 family genes are heat-responsive and high temperature-repressed genes [28]. These common temperature responsive genes could be involved in rice coping with temperature changes, and could be useful in the breeding program for rice resistant to temperature stresses.

**Figure 6:**
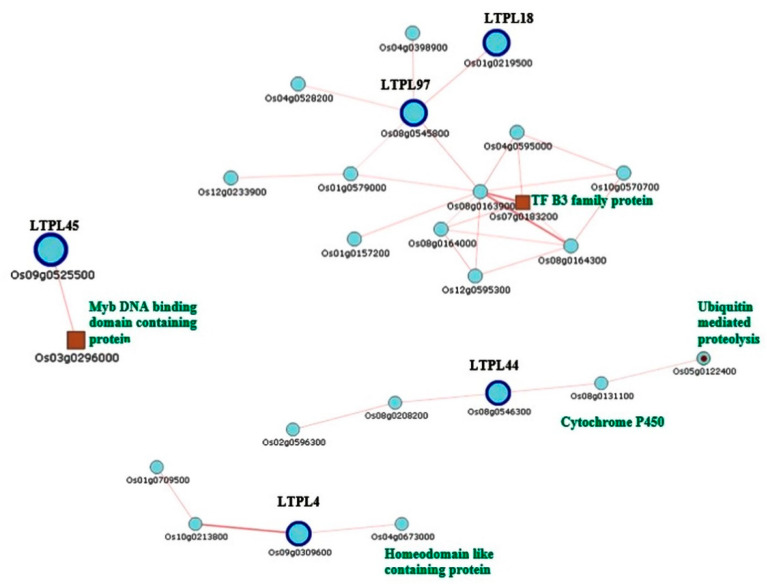
Lipid transferase protein (LTP) family genes showing coexpression with other genes.

Results from qRT-PCR expression analysis of the selected DEGs from TGMS panicles under low and high temperatures suggested that several DEGs could be TGMS-related genes controlling male reproductive development affected by temperature. All of the DEGs in the lipid binding process belong to the LTP family protein (LTPL18, LTPL57, LTPL150, LTPL44 and LTPL45). Results from our RNA-Seq analysis showed that several LTP genes (83 genes) responded to temperatures, and 11 LTPL genes were identified as our DEGs. Expression of LTP genes were reported to be required for anther development by regulating tapetum degradation of orbicules and pollen wall [48]. Lipid metabolism regulates pollen wall development [49]. The results of qRT-PCR analysis in CMS broccoli by Pei et al. [30] supported this notion. To investigate the LTP gene network, we analyzed LTP genes in a gene coexpression rice database (http://ricefrend.dna.affrc.go.jp). The result showed that some LTP genes coexpressed with many proteins in significant pathways such as transcription factors, ubiquitin proteolysis, cytochrome P450 and Myb DNA binding proteins (Figure 6). This corresponded well with our expression results, suggesting that the lipid transfer proteins and a series of related genes (TF, Ubiquitin, cytochrome P450) coordinately regulate male reproductive development in rice plants. In addition, our expression analysis by qRT-PCR suggested that three lipid transfer protein (LTP) genes; LOC_Os07g46210, LOC_Os03g24300 and LOC_Os09g13930, 2 BURP protein genes (LOC_Os09g16010 and LOC_Os04g14990) and START domain containing protein (LOC_Os06g50724) could be TGMS-related genes due to their patterns of expression in TGMS in response to temperatures that were different from those of wild-type rice plants.

BURP proteins have diverse roles in plant development and influence plant stress responses. The BURP domain was reported to be involved in cell wall localization [50]. In our study, BURP protein genes (LOC_Os09g16010 and LOC_Os04g14990) were upregulated under the low temperature (fertile) condition in the TGMS mutant, while it showed no significant difference in expression levels in low and high temperature conditions in wild-type rice plants, suggesting that the BURP genes are involved in TGMS.

Steroidogenic acute regulatory related transfer (StART) proteins or START domain containing proteins are common in plants and are predominantly found within homeodomain (HD) transcription factors. START domains are ~200 amino acid lipid/sterol binding modules [51]. The START lipid/sterol-binding module plays a role in lipid signaling in plant and is involved in intermembrane transfer of ceramide in programmed cell death [52]. Chueasiri et al. [53] reported that TGMS rice, tms2, and its RNAi-suppressed lines showed a reduced accumulation of ceramides, relative to the wild type. Our study suggested that START domain-containing protein (LOC_Os06g50724) could be TGMS related genes, probably due to its function in regulating ceramide levels involved in programmed cell death controlling pollen development.

## 4. Materials and Methods

### 4.1. Plant Materials and Growth Condition

Indica TGMS, IR68301S, rice plants obtained from the International Rice Research Institute (IRRI) were grown in control growth rooms at National Center for Genetic Engineering and Biotechnology (BIOTEC), National Science and Technology Development Agency (latitude: 14°04′50.6″ N, longitude 100° 36′09.0″ E) Pathum Thani Province, Thailand. Pregermination occurred in a tray then seedlings were transplanted into plastic pots with soil (one plant/pot). The rice seedlings were planted under natural conditions until approximately one month before flowering (70 days old). These plants were then moved into a controlled growth room with a 12-hr-light/12-hr-dark photoperiod at 26 ± 2 °C and 30 ± 2 °C for male fertile and sterile conditions, respectively. Panicles harvested from these plants were used for RNA-seq, and semithin sections were used to study anther development. For qRT-PCR expression analysis, BC3F5 (generated from IR68301S and PTT1, a Thai rice line having high yield) TGMS and wild type rice plants were grown in the same conditions with those plants used for RNA-Seq. The harvest time was during 9.00–11.00 am for all tested plants used for molecular analysis. For biological replicates, every sample for RNA isolation and semithin sections was a mixture of 10 panicles from at least five individual plants. The growth and development status of these sampled panicles were highly consistent. To observe seed setting rates, the TGMS plants were grown in natural condition. Approximately one month before flowering (70 days old), 5–10 plants (one plant/pot) were moved to grow at 24, 26, 28 and 32°C to observe their seed setting. The plants were kept in the growth room until flowering (approximately 1–1.5 month). Then they were moved back to room temperature and their seed setting were recorded.

### 4.2. Observation of Anther Development by Semithin Sections

To observe anther development by semithin sections, panicles at different developmental stages including microspore mother cell (MMC), dyad, tetrad and microspore, were harvested from the TGMS plants grown under sterile and fertile conditions. Spikelets were fixed in glutaraldehyde:paraformaldehyde (1:1) in cacodylate buffer pH 7.3. Dehydration was performed with acetone and samples were embedded in 3% toluidine blue. One to three µm thick sections were cut with an ultra-microtome JICA. Ultrastructure was observed under a stereo microscope (Olympus DP70). The facilities used for these studies were provided by Micro electron lab of Central laboratory at Kasetsart University, Kamphaeng San campus and Rice gene discovery laboratory at the National Center for Genetic Engineering and Biotechnology.

### 4.3. RNA Isolation, Library Construction and Transcriptome Sequencing

For RNA isolation, panicles of TGMS rice plants, IR68301S, grown under low (fertile) and high (sterile) temperatures were collected at the critical stage. All samples were frozen in liquid nitrogen and stored at −80 °C until use. Total RNA was extracted from panicles using TRIZOL^TM^ reagent (Invitrogen) according to the manufacturer’s instruction. The extracted RNA with the ratio of absorbance at 260 nm and 280 nm greater than 1.8 was used for transcriptome sequencing. A total of three µg of RNA treated with DNase was used for RNA sequencing. These RNA samples were sent to BGI Tech Solutions (Hong Kong) Co., Limited. ABI StepOnePlus Real-Time PCR System Agilent 2100 and Bioanaylzer were used in quantification and qualification of the sample library. The libraries were sequenced using Illumina HiSeqTM 2000 platform. The RNA-Seq data sets were submitted to NCBI. The accession number is PRJNA515791.

### 4.4. Identification of Differentially Expressed Genes, Functional Annotation and Enrichment Analysis

Illumina HiSeq paired-end raw reads were assessed for read quality using the FASTQC tool and preprocessed to trim the library adapters and low-quality bases. Estimation of gene expression (transcript abundance) from RNA-Seq data was done by Salmon program [54]. Differentially expressed genes (DEGs) between the two conditions were analyzed with the DESeq package (Version 3.3) based on the “blind” method and “fit-only” sharing mode during the data dispersion estimation step [55]. The criteria for significant differential expression with the adjusted *p* ≤ 0.05 were considered. Overall expressed genes were mapped to GO terms in the rice genome database (http://rice.plantbiology.msu.edu/downloads_gad.shtml, accessed on 8 March 2016). GO terms enrichment for the significant DEGs were analyzed by AgriGO (http://bioinfo.cau.edu.cn/agriGO/index.php, accessed on 15 March 2016) using the Singular Enrichment Analysis (SEA) tool with parameter setting as follows: statistical test method “fisher”, multi-test adjustment method “Yekutieli”, significant level “0.05”, minimum no. of mapping entries “5” and gene ontology type “complete GO”. Mapman analysis was used to perform pathway mapping of DEGs [56]. A gene coexpression network analysis was performed by the Rice Funtionally Related gene Expression Network Database (RiceFREND) version 2.0 (http://ricefrend.dna.affrc.go.jp, accessed on 28 March 2016). RiceFREND is a provided platform for identification of functionally related genes in various biological pathways and/or metabolic processes of DEGs based on microarray data. Pearson’s correlation coefficient (PCC) and mutual rank (MR) were used as correlated pairs of genes and as an index for coexpression. Heat map and hierarchical clustering of the significant DE genes were created with the BioVinci Software (BioTuring, San Diego, CA, USA) based on TPM (transcript per million) values compared between sterility and fertility samples.

### 4.5. Gene Expression Using Quantitative Real-Time RT-PCR (qRT-PCR)

For validation of the gene expression, based on DEGs analysis, at *p* ≤ 0.05 used as a threshold to judge the significance of gene expression, ten DEGs were selected for validation using RNA isolated from panicles of TGMS plants grown under low (fertile) and high (sterile) temperatures using quantitative real-time RT-PCR (qRT-PCR). To identify common temperature responsive genes and TGMS-related genes, 20 DEGs from the enriched pathways and potential functional importance were selected for qRT-PCR expression analysis using RNA samples isolated from panicles of BC3F5 TGMS plants (generated from IR68301S and PTT1, a Thai rice line having high yield) and its wild type plants to reduce background effects. cDNA was synthesized using a RevertAid First Strand cDNA Synthesis Kit (Thermo Scientific™) following the manufacturer’s instruction. Gene-specific primers were designed using Nipponbare reference genome obtained from GRAMENE database (http://www.gramene.org, accessed on 16 September 2016) and NCBI/Primer-BLAST (https://www.ncbi.nlm.nih.gov/tools/primer-blast/, accessed on 16 September 2016). Sequences of primers specific for each DEG used for qRT-PCR are listed in Table S2. The qRT-PCRs were performed in 96-well plates using the Bio-RAD iCycler iQ5 Machine (BioRad). The qPCRBIOSyGreen Mix Lo-ROX (PCR Biosystems) assay was used in a total volume of 10 µl per reaction. Each reaction mixture contained 1 µl of cDNA, 2µl of distilled water, 1 µl (10 µM) forward primer, 1 µl (10 µM) reverse primer and 5 µl of qPCRBIO SyGreen Mix. All qRT-PCR plates were carried out with following cycling conditions: 95 °C for 3 min, following 35 cycles of 95 °C for 30 s, 57 °C for 30 s and 72 °C for 30 s, then 95 °C for 1 min and a melting curve from 60 °C to 95 °C in 0.5 °C increments. Actin was used as an internal control. Triplicates were performed for all reactions. The 2−ΔΔCt method was used to calculate relative gene expression [57].

## 5. Conclusions

TGMS rice is sterile at high temperature and fertile at low temperature conditions. Results from thin sections showed that the dyad stage is a critical event controlling fertility under temperature changes in anthers of a TGMS rice line, IR68301S, suggesting that the genes involved in male reproductive development that are affected by temperature may play a role during postmeiotic microspore development. RNA-Seq analysis was used to gain more information on the genes involved in temperature-dependent rice male development, using panicles at dyad stage of IR68301S grown under high temperature (sterile) and low temperature (fertile) conditions. Two hundred and thirty-two DEGs out of 42,350 expressed genes were identified, suggesting that a small number of genes respond to temperature alteration and affect male development in rice. Results from qRT-PCR expression analysis of the 20 selected DEGs using panicles of TGMS and wild type rice plants grown under high and low temperatures showed that four4 cytochrome P450 genes, 2 LTPL, ubiquitin, aspartic protease, peptidase, jacalin-like lectin domain, WRKY, protease, and kinase had similar expression patterns under temperature alteration in both TGMS mutant and wild-type plants, suggesting that they are common temperature-responsive genes. These genes could be involved in rice coping with temperature changes, which could be useful in the breeding programs for rice resistant to temperature stresses. In addition, results from the qRT-PCR expression analysis also indicated that several DEGs including three lipid transfer protein (LTP) genes, two BURP protein genes and START domain containing protein, could be TGMS-related genes controlling temperature-dependent male reproductive development causing the TGMS phenotype. These genes could be useful for hybrid breeding programs to increase rice yield.

## Figures and Tables

**Table 1:** Seed setting rate (%) of the thermosensitive genic male sterility (TGMS) line, IR68301S, grown under controlled temperatures.

Temperature (°C)	Replicates	Seed Setting RATE (%)		Max. Temp. Mean ± SD
		Mean ± SD	(Average)	
24	1	78.1 ± 2.1	75.4 ± 1.8	24.38 ±0.6
	2	72.6 ± 1.6		24.4 ±0.6
26	1	61.9 ± 6.6	64.3 ± 5.1	25.9 ± 1.4
	2	66.7 ± 3.7		26.1 ± 1.5
28	1	0.0 ± 0.0	0.0 ± 0.0	29.4 ± 0.5
	2	0.0 ± 0.0		28.28 ± 0.1
32	1	0.0 ± 0.0	0.0 ± 0.0	32.1 ± 0.4
	2	0.0 ± 0.0		

Max. Temp was an average of the highest temperatures during panicle initiation stage to flowering stage (approximately 30 days).

**Table 2:** Summary of sequencing data quality and the statistics of the transcriptome assembly.

Map to Genome	IR68301S-Fertility	Percentage	IR68301S-Sterility	Percentage
Clean reads	47,317,390	100	47,443,806	100
Total base pairs	4,258,565,100	100	4,269,942,540	100
Total mapped reads	37,921,504	80.14	39,071,301	82.35
Perfect match	26,041,749	55.04	26,434,966	55.72
Unique match	37,193,432	78.60	38,354,275	80.84
Multi-position match	728,072	1.54	717,026	1.51
Total rice loci	66,153	100	66,153	100
Total expressed loci	21,149	31.96	21,201	32.04

**Table 3:** GO enrichment analysis of 132 upregulated differential expressed genes (DEG)s.

GO term	Ontology	Description	Number in Input List	Number in BG/Ref	*p*-Value	FDR
GO:0009653	P	anatomical structure morphogenesis	14	1141	0.00000041	0.000025
GO:0048856	P	anatomical structure development	17	1665	0.00000026	0.000025
GO:0016043	P	cellular component organization	17	1935	0.0000021	0.000082
GO:0007275	P	multicellular organismal development	19	3543	0.00043	0.01
GO:0032502	P	developmental process	20	3791	0.00036	0.01
GO:0032501	P	multicellular organismal process	19	3619	0.00056	0.011
GO:0008289	F	lipid binding	5	346	0.0014	0.041

P, biological process; F, molecular function.

**Table 4:** Common temperature responsive genes and TGMS-related gene.

Gene	Description
	**Common Temperature Responsive Genes**
*LOC_Os01g50410*	STE_MEKK_ste11_MAP3K.6—STE kinases include homologs to sterile 7, sterile 11 and sterile 20 from yeast, expressed
*LOC_Os11g04010*	ICE-like protease p20 domain containing protein, putative, expressed
*LOC_Os02g08440*	WRKY71, expressed
*LOC_Os01g24710*	jacalin-like lectin domain containing protein, expressed
*LOC_Os01g37000*	carboxyl-terminal peptidase, putative, expressed
*LOC_Os02g48870*	aspartic proteinase nepenthesin-2 precursor, putative, expressed
*LOC_Os09g32020*	ubiquitin fusion degradation protein, putative, expressed
*LOC_Os01g12020*	LTPL18—Protease inhibitor/seed storage/LTP family protein precursor, expressed
*LOC_Os09g35700*	LTPL45—Protease inhibitor/seed storage/LTP family protein precursor, expressed
*LOC_Os04g40470*	cytochrome P450, putative, expressed
*LOC_Os01g72270*	cytochrome P450, putative, expressed
*LOC_Os08g05620*	cytochrome P450, putative, expressed
*LOC_Os01g63540*	cytochrome P450, putative, expressed
*LOC_Os01g50750*	zinc finger, C3HC4 type domain containing protein, expressed
	**TGMS** **-related gene**
*LOC_Os09g16010*	BURP domain containing protein, expressed
*LOC_Os06g50724*	START domain containing protein, expressed
*LOC_Os07g46210*	LTPL2—Protease inhibitor/seed storage/LTP family protein precursor, expressed
*LOC_Os03g24300*	LTPL1—Protease inhibitor/seed storage/LTP family protein precursor, expressed
*LOC_Os09g13930*	LTPL4—Protease inhibitor/seed storage/LTP family protein precursor, expressed
*LOC_Os04g14990*	BURP domain containing protein, expressed

## Data Availability

The RNA-Seq data sets were submitted to NCBI. The accession number is PRJNA515791 (https://www.ncbi.nlm.nih.gov/bioproject/PRJNA515791/).

## Supplementary Materials

The following are available online at https://www.mdpi.com/article/10.3390/plants10040663/s1; Table S1: Primers of differentially expressed genes (DEGs) used for qRT-PCR.
