# Supplementary material for: RNA Sequencing Reveals Rice Genes Involved in Male Reproductive Development under Temperature Alteration
Source: Plants (Basel). 2021 Mar 30;10(4):663. doi: 10.3390/plants10040663 (PMC8066911; doi:10.3390/plants10040663)
Supplement: Supplementary file 1 [file plants-10-00663-s001.pdf]

Table S1. Primers of differentially expressed genes (DEGs) used for qRT-PCR.

DE gene	Forward primers (5´–3´)	Reverse primers (5´–3´)
LOC_Os01g50410	ACGAAGGATGGATCGAGGTG	CGATCGCTTCTGCTTCATTG
LOC_Os11g04010	TGAGCGATCAGGGGTTCGAG	CCGCGGTAACCGTCTTCTTCT
LOC_Os04g14990	CTCCGGCTCAGGAGTACACG	GTCTTTGCCGACCAACGACG
LOC_Os07g46210	CATTCCTCGTGAAGCCGCAG	GAAGTGTTAAGCGACTGGTGGT
LOC_Os09g16010	GCCGAGTCGTTCAGGACGTA	GACGTGAAGCTGTCGTTGCC
LOC_Os02g08440	TGCAAGCCCAAGATCTCCAAGC	GTGTTGTCCTCCGCGCTTCTC
LOC_Os06g50724	GATCACAGGATGGGGAAGGA	CAACCGGTCACTTTGGATGA
LOC_Os01g50750	ATCTTCCTGAGGCGGGTGTA	AGTAGAGGAGGGGGAGGCTG
LOC_Os01g24710	CACCCAAGAAGCTGTTAGGC	GTCTTGCAGTGGAATGCTGA
LOC_Os01g37000	TCCTCCTGGTGGCTCTCTTC	GATGGTCGAATGCAGGTTGA
LOC_Os02g48870	CAGCAGTCGAAGGTGTCGTC	GCCCTGTAGGTGCTCGACTT
LOC_Os09g13930	CACCATTGCCACCACTGTCATC	GGCCTCGTTTGCCAGGTTGA
LOC_Os09g35700	GGCGATGACGATGACGATG	TGTTGATGATGTCGAGCGTG
LOC_Os03g24300	GCGAGGCGAATGCAAGCTAC	AGCTGATAGTCAAGGACCTCATCAT
LOC_Os01g12020	TGTCATCCTGCATGCGTGT	TTGCAAAGCTCGTTTGGGTT
LOC_Os01g63540	TCCATCAAGTACCGCCTGTG	AACAAGCTCGGGAACAGGAA
LOC_Os04g40470	TCCTACGGCCAGAAGAGCAT	AGAGCCTGGGTCTCGTTGAG
LOC_Os09g32020	ATTTCGCACCTCCACTCGAT	CCTGATGCTGCTGTCTCCTG
LOC_Os01g72270	TTCTTCGCGGCGTTCGATGA	CCGTGAAGCGTGACAGCAAG
LOC_Os08g05620	AACACGCCGATGAACTTCGC	AACTCCCAGACGAGGTTGGC
